# KIF2C Facilitates Tumor Growth and Metastasis in Pancreatic Ductal Adenocarcinoma

**DOI:** 10.3390/cancers15051502

**Published:** 2023-02-27

**Authors:** Xing Huang, Feng Zhao, Quan Wu, Zitong Wang, Haiyue Ren, Qiqi Zhang, Zhe Wang, Jin Xu

**Affiliations:** 1 Department of General Surgery, Shengjing Hospital, China Medical University, Shenyang 110004, China; 2 Department of Stem Cells and Regenerative Medicine, China Medical University, Shenyang 110122, China; 3 Department of Pathology, Shengjing Hospital, China Medical University, Shenyang 110004, China

**Keywords:** KIF2C, PDAC, prognosis, invasion, migration, proliferation, cell cycle

## Abstract

**Simple Summary:**

For patients with pancreatic cancer, due to the concealment of early symptoms, rapid progress, and easy metastasis, the prognosis is grim. Based on clinical specimens, we found that KIF2C is abnormally expressed in pancreatic cancer, related to the stage of the patient. Through in vivo and in vitro experiments, we confirmed that KIF2C affects the proliferation, invasion, and metastasis of pancreatic cancer. Following this, via flow cytometry, we detected that KIF2C affects the cell cycle of pancreatic cancer and verified the expression of some genes related to the underlying mechanism in the sequenced transcriptome data.

**Abstract:**

Pancreatic ductal adenocarcinoma (PDAC) is a highly lethal cancer with a poor prognosis. For PDAC, an increase in the survival time of patients and a reduction mortality have not yet successfully been achieved. In many research works, Kinesin family member 2C (KIF2C) is highly expressed in several tumors. Nevertheless, the role of KIF2C in pancreatic cancer is unknown. In this study, we found that KIF2C expression is significantly upregulated in human PDAC tissues and cell lines such as ASPC-1 and MIA-PaCa2. Moreover, KIF2C upregulation is associated with a poor prognosis when combining the expression of KIF2C with clinical information. Through cell functional assays and the construction of animal models, we showed that KIF2C promotes PDAC cell proliferation, migration, invasion, and metastasis, both in vitro and in vivo. Finally, the results of sequencing showed that the overexpression of KIF2C causes a decrease in some proinflammatory factors and chemokines. The cell cycle detection indicated that the pancreatic cancer cells in the overexpressed group had abnormal proliferation in the G2 and S phases. These results revealed the potential of KIF2C as a therapeutic target for the treatment of PDAC.

## 1. Introduction

Pancreatic cancer remains a highly malignant tumor, and the survival rate of diagnosed patients is less than 10% [1]. Due to the difficulty in the early diagnosis of pancreatic cancer and the limited treatment options after diagnosis, PDAC is projected to become the second leading cause of cancer deaths by 2040 [1]. In recent decades, even though a lot of research has been conducted on the pathogenesis of pancreatic cancer, the specific molecular mechanism is not completely clear, and the prognosis of patients is still grim. Surgical operation is the most thorough treatment for pancreatic cancer, but fewer than 20% of patients have the opportunity to undergo surgical resection, and 80% of these patients relapse after surgery [2]. Although some research related to pancreatic cancer has shown that immune checkpoint blockades can improve the survival rate of patients to a certain extent, they do not have a profound impact on the overall survival time and reduce the postoperative recurrence rate [3,4,5]. Therefore, there is still an urgent need to identify novel therapeutic targets and further explore the underlying mechanisms.

Mitosis is a dynamic process of cell division, which is strictly regulated [6]. In this process, the chromosomes of cells are replicated by the separation of spindles with a microtubule structure, composed of dynamic polymers of α and β tubulin [7]. This structure is regulated by a series of kinases, motor proteins, and microtubule-associated proteins [8,9,10]. The kinesin 13 family is one of these regulators, which plays a vital role in regulating spindle assembly, chromosome aggregation, and segregation [11,12,13,14]. KIF2C (mitotic centromere-associated kinesin, MCAK) is a member of the kinesin family [15]. It is an efficient depolymerase, has a significant affinity for the end of the microtubule, and can effectively realize the depolymerization and recombination of the end of the microtubule. KIF2C can improve the depolymerization effect and the specificity of the binding tubulin terminal by acting as a dimer [16,17,18,19]. Additionally, the precise regulation of KIF2C not only ensures the normal physiological process of cell mitosis but also interferes with the function of KIF2C, which may defect mitosis and make the structure of the chromosome unstable, both signs of tumor progression [9]. It is also involved in the remodeling of the cytoskeleton during metastasis and invasion, which is related to the abilities of tumor invasion and metastasis [20,21,22]. KIF2C has been reported to be involved in the proliferation and migration of hepatocellular carcinoma and breast cancer [23,24]; however, no studies have shown that KIF2C is related to the occurrence and development of pancreatic cancer. It is worth noting that there is sufficient evidence to indicate that the expression of KIF2C is abnormal in PDAC and plays a role in tumor progression.

In this study, we report that the expression of KIF2C in pancreatic cancer is increased, and it is closely connected to the tumor stage and prognosis of patients. Both in vitro and in vivo experiments show that the expression level of KIF2C affects the invasion, metastasis, and proliferation of pancreatic cancer.

## 2. Materials and Methods

### 2.1. Survival Curve and Correlation Analysis

The gene expression profile Interactive Analysis browser (Accessed on 5 July 2021, http://gepia.cancer-pku.cn/index.html) is a web-based tool for analyzing the data provided by gene expression profiles and gene tissue expression. The disease-free and overall survival curves associated with KIF2C were obtained from a GEPIA online analysis. In addition, the expression of KIF2C in different tumors was learned through a GEPIA analysis, as well as the expression differences between pancreatic cancer and adjacent cancer, which laid a certain foundation for future work. The correlation between KIF2C and CDC20 was also analyzed by GEPIA. The OS, PFS, and GSEA enrichment analyses were analyzed by R studio from TCGA datasets.

### 2.2. Patients and Specimens

The 14 pairs of cancer and paracancerous tissues used in the qPCR analysis were all from Shengjing Hospital, Shenyang, China. The paraffin-embedded pathological specimens used for the immunohistochemical analysis from 200 patients with PDAC were collected from the archives of the Department of Pathology, Shengjing Hospital, between January 2017 and August 2019. These cases were randomly selected surgical slides from different inpatient wards, and the pathological information was completed by telephone follow-up at the same time as the completion of the immunohistochemistry. Patient consent was waived due to no additional inspection or injury, and all specimens used in the study were approved by the Committees for Ethical Review of Research Involving Human Subjects in the China Medical University.

### 2.3. Immunohistochemical (IHC) Staining

Briefly, all of the steps of the immunohistochemistry were performed according to the instructions of the Immunohistochemical Kit (UltraSensitive^TM^ SP IHC Kit, MXB, China). We set the depth of staining as 0–3 (0 = none, 1 = weak, 2 = medium, and 3 = strong). The staining range was divided into the five grades of 0–4 (0 = none, 1 = <25%, 2 = 25–50%, 3 = 50–75%, and 4 = >75%). KIF2C expression = staining depth * staining range.

### 2.4. Cell Culture

ASPC-1 and MIA-PaCa2 were purchased from Procell (Wuhan, China). All cell lines were authenticated. ASPC-1 was cultured in 1640 (VivaCell, Shanghai, China) with 10% 30070 (Hyclone, Logan, UT, USA), and MIA-PaCa2 was cultured in high-glucose Dulbecco’s modified Eagle’s medium (DMEM; VivaCell, Shanghai, China) with 10% 30070 and 3% horse serum (Gibco, Grand Island, New York City, NY, USA). Both of the cell lines were cultured in a 37 °C humidified incubator (Thermo, Waltham, MA, USA) with a 5% CO_2_ environment.

### 2.5. Plasmid Construction and Transfection

All the plasmids were purchased from GenePharma (Suzhou, China). The sequence of shRNA and siRNA was 5′-GCATAAGCTCCTGTGAATATA-3′. Cell transfection was performed using Lipofectamine 2000 Reagent (11668-019, Invitrogen, Carlsbad, CA, USA) following the manufacturer’s instructions. All transfection efficiencies were verified by qPCR after 24 h. shRNA and overexpressed plasmid transfection were screened with g418 (Ig0010, Solarbio, Beijing, China). Since the plasmid contained g418 resistance, 600, 800, and 1000 μg/mL of g418 antibiotics were added to the transfected six-well plate once every three days for a week. Then, the dose of antibiotics was halved and maintained for a month. The transfection efficiency was detected by qPCR. The cells selected by the 800 μg/mL concentration of antibiotics had the highest transfection efficiency. The cells were subcultured in a T75 culture flask (Thermo, Waltham, MA, USA) and frozen.

### 2.6. Cell Proliferation, Migration, and Invasion Assays

The cell proliferation experiment was completed by the MTT and soft agar colony formation assays. MTT: The cells were seeded in a six-well plate for transfection, and within 24–48 h of transfection, the cells were digested, resuspended, and counted. Then, 5000 cells were evenly plated in a 96-well plate, and 20 μL of MTS (5 mg/mL) were added every day to incubate for 2 h, and the absorbance was measured. There were three wells in each group, and the cell proliferation was measured continuously for four days. Soft agar colony formation assays: Firstly, 1.2% agarose was mixed with 20% 30070 + 2*1640 medium/high-glucose DMEM in a 1:1 ratio, and 1.5 mL of the mixture was added to each well of the six-well plate, gently mixed, and left to set at room temperature (no bubbles were generated). Secondly, 0.7% agarose was mixed with 20% 30070 + 2*1640/high-glucose DMEM in a 1:1 ratio, and cell suspension prepared in advance was added into the mixture, which was quickly mixed and added into the six-well plate (1 mL per well). After the upper layer solidified, it was placed in the incubator at 37 °C for 2–3 weeks. Add 10% 30070 + 1640/high-glucose DMEM every two days to prevent excessive drying. The Transwell migration and Matrigel (Corning, Corning, New York City, NY, USA) invasion assays were mainly performed as below. The main process can be briefly described as follows: First, the Matrigel was diluted with serum-free medium (1:9), the mixture was evenly dripped to the upper part of the chambers (50 μL per chamber), and then, the 24-well plate was placed in a 37 °C humidified incubator for 4 h. Cells (5*10^4^) ASPC-1/MIA-PaCa2 (100 μL) and 600 μL of 1640 medium (ASPC-1 cell line) or high-glucose DMEM cell (MIA-PaCa2 cell line) suspension were added to the upper layer of the chamber, and 600 μL of the ASPC-1 cell line culture medium (1640 with 10% 30070) or MIA-PaCa2 cell line culture medium (high-glucose DMEM with 10% 30070 and 3% horse serum) were added to the lower layer. When the cells were placed in the chamber for approximately 12 h, they were fixed with methanol, stained with hematoxylin and eosin, dried, and photographed under a microscope to observe the migration ability. For the Matrigel invasion, the migrated cells were fixed with methanol and stained with hematoxylin and eosin after 36 h. The data were all obtained from three independent experiments.

### 2.7. RNA Isolation and Quantitative RT-PCR

All the tissues and cell RNA were obtained via Trizol (9109, TaKaRa, Shiga, Japan) extraction. The extracted RNA was reverse-transcribed using a PrimeScript RT-PCR Kit (RR047A, Takara, Shiga, Japan) to obtain cDNA. A SYBR GreenPCR Kit (RR820A, TaKaRa, Shiga, Japan) was the only reagent used to conduct qRT-PCR. The primer sequences can be found in Supplementary File S2.

### 2.8. Western Blot

Cell and tissue samples were cleaved in a cold RIPA cleavage buffer (Beyotime, Shanghai, China). Determination of the protein concentration was achieved using a BCA Analytical Kit (Beyotime, Shanghai, China). The proteins were layered by electrophoresis through 10% SDS-PAGE gel (Beyotime, Shanghai, China) and then transferred to 0.22 μm PVDF membranes. The protein-transferred PVDF membrane was blocked with 5% skimmed milk powder for 2 h and subsequently washed three times with TBST, each for 5 minutes, before being incubated with the primary antibody overnight at 4 °C. The primary antibody was recycled, the membrane was washed with TBST three times (each for 5–10 minutes), and then, the secondary antibody was applied for 1 h at room temperature. After three washes with TBST, the ECL (Beyotime, Shanghai, China) was used to visualize the PVDF membrane. For quantification, the optical density of individual bands was analyzed by ImageJ software (V1.8.0, NIH, USA), and the values were normalized to GAPDH (1:5000, Proteintech, Chicago, USA). Full Western blot images can be found in Supplementary File S1.

### 2.9. Antibodies

The antibodies used in this study were as follows: KIF2C (1:1000, Sigma, Darmstadt, Germany), IL-1β (1:1000, Elabscience, Wuhan, China), IL-18 (1:1000, Elabscience, Wuhan, China), CDC20 (1:2000, Proteintech, Chicago, USA), and GAPDH: (1:5000, Proteintech, Chicago, USA).

### 2.10. Animals Model

Five-week-old female BALB/c nude mice were used in this study. In the subcutaneous xenograft model, MIA-PaCa2 cells were digested, resuspended with PBS, and counted. All 2*106 MIA-PaCa2 cells were resuspended with 100 microliters of bovine serum albumin solution and injected into the axilla of each nude mouse in the tumorigenesis experiment. The tumor formed two weeks after the injection, and the diameter of the tumor was measured every three days. Two weeks later, the nude mice were sacrificed, and the tumors were harvested, weighed, fixed, and paraffin-embedded for further analysis. For the metastatic assay, 2*106 MIA-PaCa2, the mice were euthanized and the lungs were excised and embedded in paraffin for further analysis.

### 2.11. Embedding and Slicing

After the lung tumorigenesis model of the nude mice was taken out, the lungs were immediately squeezed in formalin (Beyotime, Shanghai, China) to fill the lungs. After soaking for one day, they were embedded in OCT (Tissue-Tek^®^ O.C.T. Compound, Sakura Finetek, Torrance, CA, USA) and preserved at −80 °C for frozen sections. The embedded tissue was sliced with a frozen slicer (CM1950, Leica, Weztlar, Germany) with a thickness of 8 microns.

### 2.12. Cell Cycle Detection

All operations were carried out in accordance with the instructions (Cell Cycle and Apoptosis Analysis Kit, Beyotime, Shanghai, China).

### 2.13. H&E Staining

For hematoxylin–eosin staining (Modified Hematoxylin–Eosin (HE) Stain Kit, G1121, Solarbio, Beijing, China), we circled all of the tissues on the slices with an immunohistochemical pen (YA0310, Solarbio, Beijing, China) and then inserted all of the slides into the slice rack before rinsing with tap water for 10 min. Then, we took out the slides, put them on the dye board, and covered the tissue with drops of hematoxylin solution for 15 min before rinsing with tap water for 10 s. Next, we covered the tissue with drops of differentiation solution for 5 s before rinsing with tap water for 30 s. Following this, we covered the tissue with drops of bluing solution for 1 minute before rinsing with tap water for 30 s. Lastly, we covered the tissue with drops of eosin solution for 30 s before rinsing with tap water for 5 s. The slices were then dehydrated in a concentration of 75%, 85%, 95%, or 100% ethanol for 3 s; anhydrous ethanol II for 1 minute; and xylene I and II and each transparent for 1 minute. Finally, the slices were sealed with xylene and neutral balsam (G8590, Solarbio, Beijing, China) (1:1).

### 2.14. Statistical Analysis

One-way ANOVAs were used to compare the expression levels of KIF2C or other targets among three or more groups, while *t*-tests were used to compare the expression levels of KIF2C or other targets between two groups. In order to compare the OS of the patients between subgroups, we used the Kaplan–Meier method and bilateral logarithmic rank tests.

IBM SPSS 26 software (SPSS V26.0, Chicago, IL, USA) and Prism 9 (GraphPad Software V9.0, CA, USA) were used to analyze the experimental data. All experiments were carried out at least three times; the data are expressed as the mean ± standard deviation. The difference was statistically significant at *p* < 0.05.

### 2.15. Data Availability Statement

The original contribution proposed in this study is included in the article/supplementary materials. Further inquiries can be directed to the corresponding author.

### 2.16. Ethics

This project was approved by the Institutional Review Committee of Shengjing Hospital of China Medical University (ethical approval codes: 2021PS424K and 2021PS747K).

## 3. Results

### 3.1. Upregulation of KIF2C in PDAC

**Figure 1:**
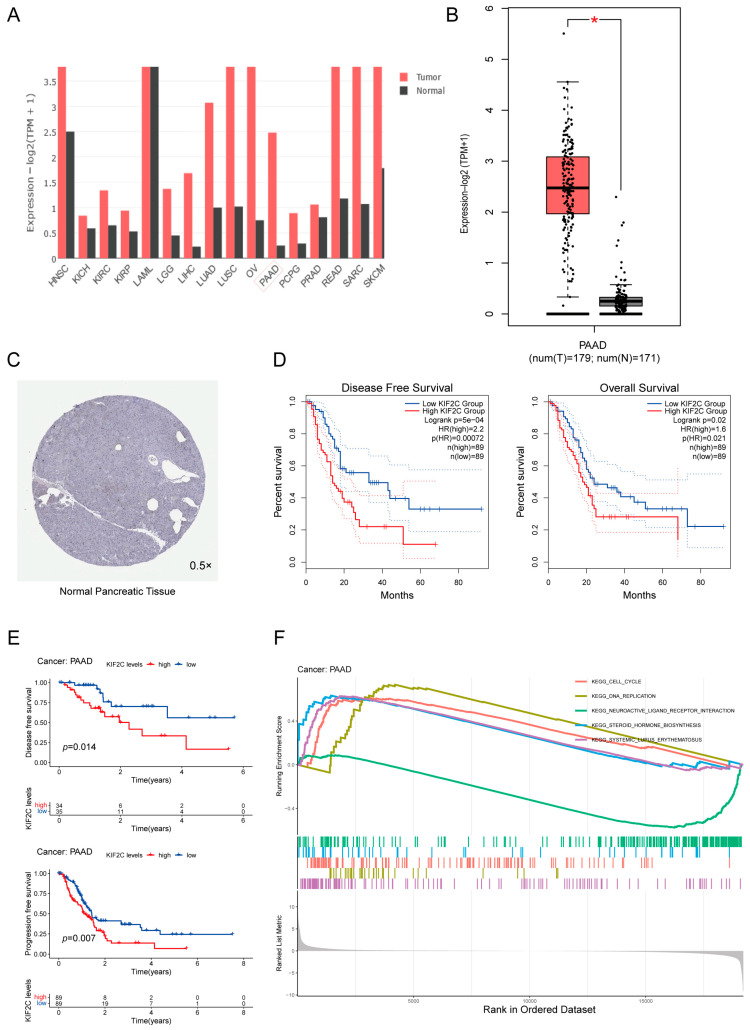
The expression level of KIF2C in PAAD and its relationship with survival and prognosis. (**A**) Expression levels of KIF2C in different tumors in the TCGA database. (**B**) The expression of KIF2C in PAAD was significantly higher than that in normal tissues in the TCGA database (Num(T) = 179, Num(N) = 171, *p < 0.05). (**C**) The expression of KIF2C in the pancreas from The Human Protein Atlas. (**D**) Kaplan-Meier curves for the OS (HR(High) = 1.6) and DFS (HR(High) = 2.2) of patients in the high-and low-KIF2C groups. (**E**) Kaplan-Meier curves for the PFS and DFS of patients in the high-and low-KIF2C groups. (**F**) Cell cycle, steroid hormone biosynthesis, systemic lupus erythematosus, and DNA replication were related to KIF2C in the GSEA enrichment analysis.

The results of the TCGA analysis indicated that the expression of KIF2C was different in various cancers (Figure 1A). The expression level of KIF2C in pancreatic cancer was also higher than that in normal tissues (Figure 1B). Based on the data of The Human Protein Atlas, we found that, in normal pancreatic tissue, KIF2C was lowly expressed in exocrine glandular cells, while KIF2C was not detected in endocrine cells (Figure 1C). In other words, the expression of KIF2C in normal pancreatic tissue is low, but after normal tissue develops into cancer, its expression increases rapidly. This means that KIF2C may be an important protein in the occurrence and development of pancreatic cancer. The difference in the expression of KIF2C in pancreatic cancer makes us think about the relationship between KIF2C and survival. The Kaplan–Meier analysis of the TCGA database showed that the expression of KIF2C was closely related to the survival time of patients; in particular, the high expression of KIF2C had a deeper impact on disease-free survival (Figure 1D). In order to better determine the impact of KIF2C on survival, we analyzed other datasets of TCGA and obtained the same results (Figure 1E). KIF2C affects the progression-free, disease-free, and overall survival. Moreover, the GSEA enrichment analysis indicates that KIF2C is closely associated with the cell cycle, DNA replication, etc. (Figure 1F).

### 3.2. Abnormal Expression of KIF2C in Clinical Specimens

**Figure 2:**
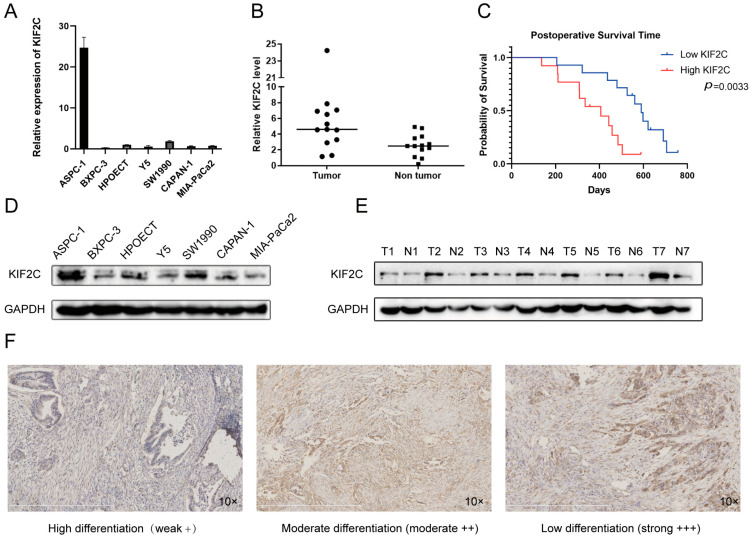
Immunohistochemical results of KIF2C in pancreatic carcinoma with different differentiation. (**A**) Real-time PCR detection of the KIF2C expression in different pancreatic cancer cell lines. (**B**) Real-time PCR detection results of the KIF2C expression in 13 pairs of fresh specimens (pancreatic cancer and paired adjacent cancers). (**C**) Kaplan–Meier curves for the postoperative survival of patients in the high- and low-KIF2C groups. (**D**) Western blot of the KIF2C expression in different pancreatic cancer cell lines. (**E**) Western blot of the KIF2C expression in 7 pairs of fresh specimens (pancreatic cancer and paired adjacent cancers). (**F**) Immunohistochemical staining intensity of KIF2C in different tumor differentiation grades.

The results of the bioinformatics analysis aroused our interest. We noticed that there is no relevant literature to show whether the difference in KIF2C expression has an influence on pancreatic cancer. At first, we performed qPCR detection and a Western blot of the KIF2C expression in the cell lines, including ASPC-1, BXPC-3, HPOECT, Y5, SW1990, CAPAN-1, and MIA-PaCa2. It was found that the expression of KIF2C in the ASPC-1 cell line was obviously increased in comparison to the expression in the other cell lines (Figure 2A,D). After validating the cell lines, we extracted the cancer and paired adjacent cancer RNAs from pancreatic cancer patients and detected the relative expression of KIF2C by qPCR after reverse transcription (Figure 2B). After that, we also extracted proteins from cancer and adjacent tissues and detected the expression of KIF2C by Western blot (Figure 2E). We intuitively found that the expression of KIF2C in cancer tissue was higher than that in adjacent tissue. Next, we carried out immunohistochemical staining on 70 randomly selected pancreatic cancer specimens. By analyzing the depth and range of staining, the expression level of KIF2C in pancreatic cancer was prominently higher than that in normal or highly differentiated tissues (Figure 2F). At the same time, after selecting the pathological tissues to be stained, we collected the relevant clinical information of the patients through case files and follow-up. By combining the staining results with the clinical information of the specimens, we acquired a meaningful result (Table 1). Patients with advanced TNM pancreatic cancer had higher KIF2C expression levels compared to patients with early TNM. In addition, KIF2C was also connected to the degree of tumor differentiation, and the expression of KIF2C was significant in poorly differentiated tumors. Furthermore, a combined analysis of KIF2C expression and the postoperative survival time showed that patients with high KIF2C expression had a shorter survival time than those with low KIF2C expression (Figure 2C).

### 3.3. Knockdown of KIF2C Inhibits PDAC Cell Proliferation, Migration, and Invasion In Vitro

**Figure 3:**
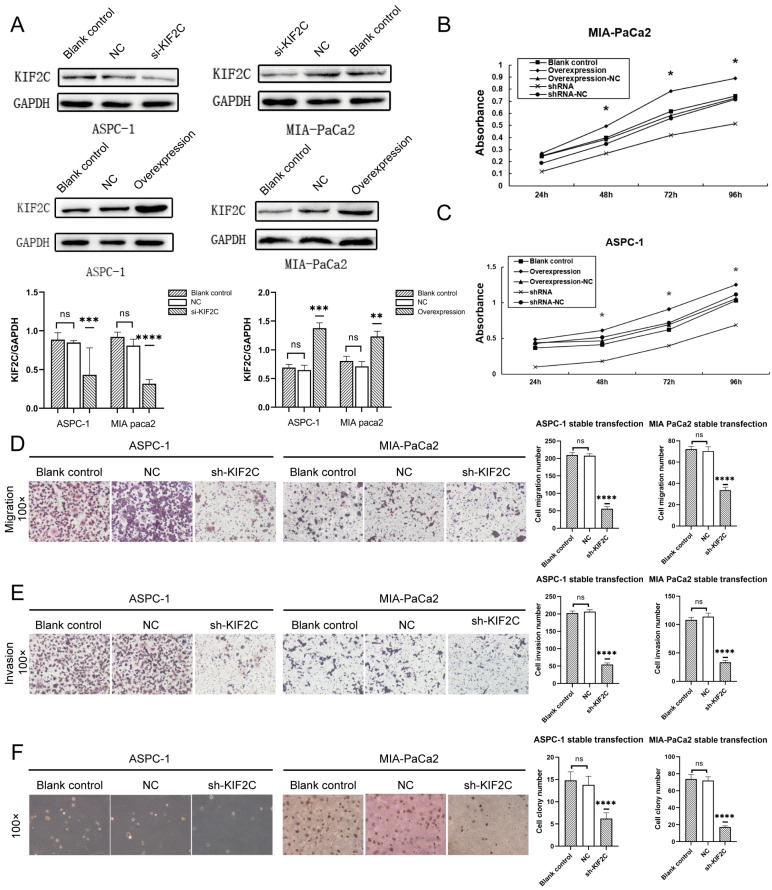
A series of assays on the invasion, migration, and proliferation of the transfected cell lines (ASPC-1 and MIA-PaCa2). (**A**) Western blot of the blank control, NC, overexpression, and si-KIF2C groups (** *p* < 0.01, *** *p* < 0.001, **** *p* < 0.0001). (**B**) A line chart of the absorbance for the MTT assay on ASPC-1 (* *p* < 0.05). (**C**) A line chart of the absorbance for the MTT assay on MIA-PaCa2 (* *p* < 0.05). (**D**) Migration assay on ASPC-1 and MIA-PaCa2 stable transfection. On the right is a statistical bar chart (**** *p* < 0.0001). (**E**) Invasion assay on ASPC-1 and MIA-PaCa2 stable transfection. On the right is a statistical bar chart (**** *p* < 0.0001). (**F**) A soft agar colony formation assay on ASPC-1 and MIA-PaCa2 stable transfection. On the right is a statistical bar chart (**** *p* < 0.0001).

Subsequently, we investigated whether KIF2C affects the proliferation, migration, and invasion of PDAC cells. First, we altered the KIF2C expression in APSC-1 and MIA-PaCa2 cells by the transfection of overexpressed plasmid and siRNA (Figure 3A). In order to obtain stably expressing cells, we performed g418 antibiotic screening for one month after the transfection of overexpression and shRNA plasmids. As for the MTT assay, we plated 5000 cells per well evenly in 96-well plates to detect proliferation in the different treatment groups. The absorbance of three wells in each group was measured by the microplate reader every day for four consecutive days, and a line graph was made after taking the average value (Figure 3B,C). The line graph clearly shows that the proliferation rate of the interference group was significantly lower than that of the other groups. Normally, the proliferation rate of ASPC-1 was faster than that of MIA-PaCa2. It can be seen from the figure that the inhibition of ASPC-1 proliferation by knocking down KIF2C was stronger than that of MIA-PaCa2 at 24 and 48 h. As for the migration and invasion assays, 50,000 cells were placed in each chamber for Transwell migration and Matrigel invasion assays. After 12 h, the cells in the migration assays were fixed and stained; after 36 h, the cells in the invasion assays were fixed and stained. After gently wiping away the non-infiltrated cells from the chamber and allowing the chamber to dry, the effect of sh-KIF2C on cell migration and invasion was observed under a microscope (Figure 3D,E). Under the microscope, it was observed that, for both the ASPC-1 and MIA-PaCa2 cell lines, the interference of KIF2C caused a distinct decrease in the ability of cancer cells to invade and migrate. However, the evidence of in vivo experiments is also imperative. Before this, we chose to simulate the in vivo experiment through a soft agar colony formation assay to explore the expected results. Since the proliferation rate of MIA-PaCa2 is slower than that of ASPC-1 and the cell size of MIA-PaCa2 is also smaller, there were 5000 cells per well in a six-well plate in the ASPC-1 group and 10,000 cells per well in the MIA-PaCa2 group. In two to three weeks, the laid single cells proliferated to form a cell mass (Figure 3F). Under the microscope, in the interfered treatment group, the proliferation of cancer cells was significantly inhibited. These results suggest that KIF2C may be one of the key genes with a strong ability to proliferate and invade in pancreatic cancer.

### 3.4. Overexpression of KIF2C Promotes PDAC Cell Proliferation, Migration, and Invasion In Vitro

**Figure 4:**
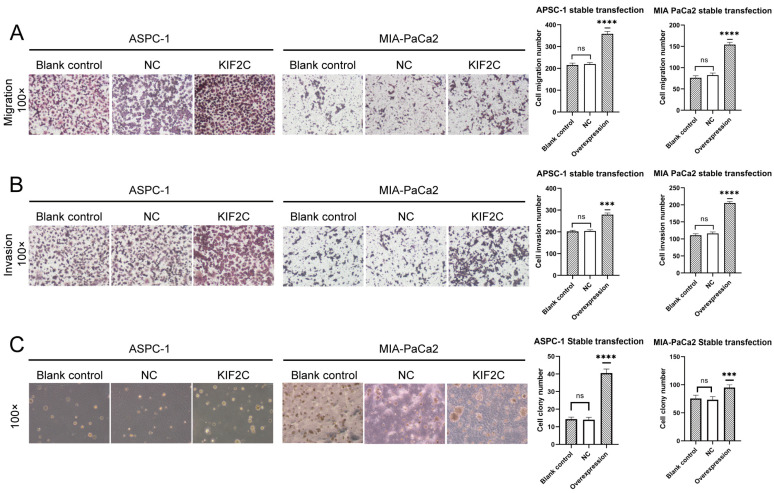
A series of assays on the invasion, migration, and proliferation of the transfected cell lines (ASPC-1 and MIA-PaCa2). (**A**) Migration assay on ASPC-1 and MIA-PaCa2 stable transfection. On the right is a statistical bar chart (**** *p* < 0.0001). (**B**) Invasion assay on ASPC-1 and MIA-PaCa2 stable transfection. On the right is a statistical bar chart (*** *p* < 0.001, **** *p* < 0.0001). (**C**) Soft agar colony formation assay on ASPC-1 and MIA-PaCa2 stable transfection. On the right is a statistical bar chart (*** *p* < 0.001, **** *p* < 0.0001).

Irrespective of the analysis of bioinformatics or the verification of clinical samples, KIF2C in pancreatic tumor cells is much higher than that in a normal pancreas. The overexpression of KIF2C is very crucial to explore whether it can promote tumor proliferation and enhance the abilities of invasion and migration. In the MTT assays, the effect of the overexpression of KIF2C on the proliferation of ASPC-1 was slightly weaker than that of MIA-PaCa2 (Figure 3B,C). However, in both cell lines, the overexpression of KIF2C did significantly promote the proliferation of tumor cells. As for the migration and invasion assays, the penetration ability of overexpressed tumor cells was stronger than that of the control group (Figure 4A,B). In the soft agar colony formation assay, it was shown that, in the group with the overexpression of KIF2C, both MIA-PaCa2 and APSC-1 formed more and larger cell colonies than those in the control group (Figure 4C). This means that the overexpression of KIF2C in pancreatic cancer is not meaningless. Pancreatic cancer patients have a low survival rate, a high degree of malignancy, and a high metastasis rate. KIF2C may play a promoting role in these aspects.

### 3.5. KIF2C Promotes PDAC Cell Proliferation, Migration, and Invasion In Vivo

**Figure 5:**
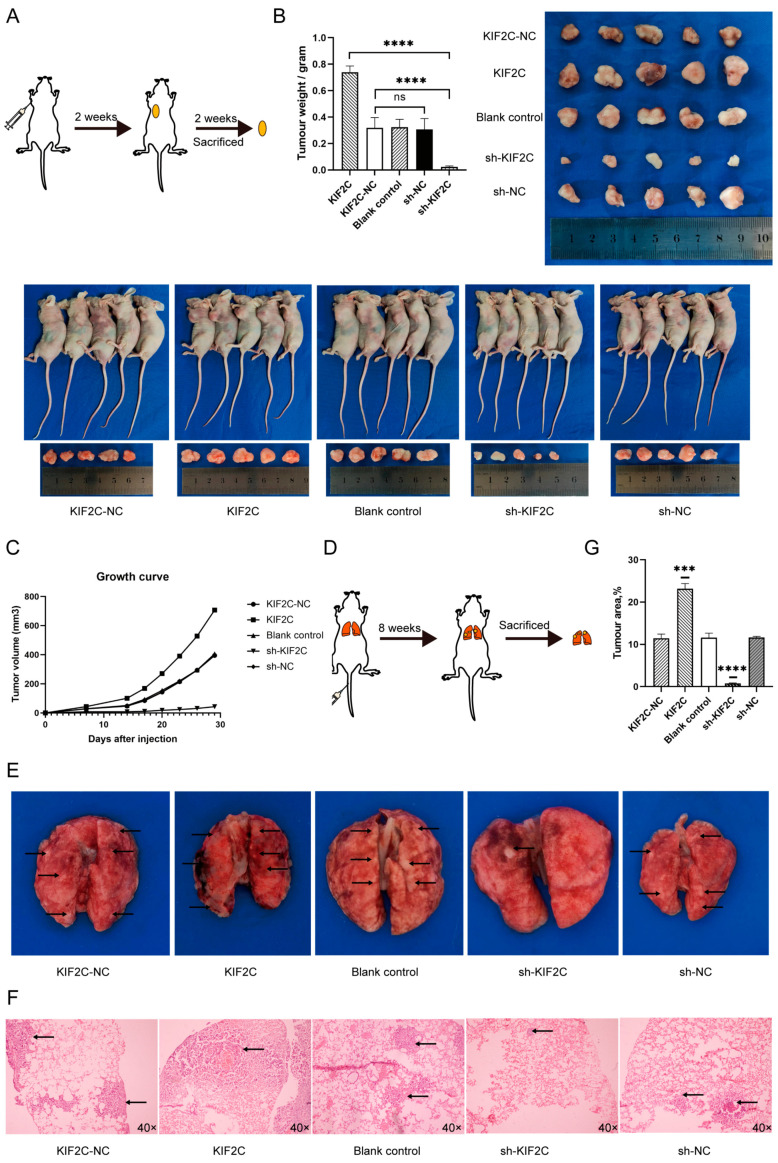
Subcutaneous and pulmonary tumorigenesis assay in vivo. (**A**) A model picture of subcutaneous tumorigenesis. (**B**) The size and weight of the subcutaneous tumor in the different groups (**** *p* < 0.0001). (**C**) Growth changes of subcutaneous tumorigenesis at 0–4 weeks in each group. (**D**) A model picture of lung metastases. (**E**) Lung tissue with tumor metastasis in the different groups. (**F**) H&E staining of lung metastasis under a microscope in the different groups. (**G**) Lung metastasis area was measured in each group (*** *p* < 0.001, **** *p* < 0.0001).

With the strong support of the above in vitro experiments, we further explored whether KIF2C has the same effect in vivo. We established a model of ectopic pancreatic cancer by subcutaneous injection in female nude mice. Our team chose to use MIA-PaCa2 when building a subcutaneous pancreatic cancer tumor model, because in previous experiments, we observed that the animal model built by ASPC-1 causes the tumor to rupture and bleed due to vascular infiltration, among other reasons. Two weeks later, tumor formation was observed, and the tumor diameter was measured every three days. Two weeks later, the nude mice were sacrificed, and the tumor was measured to compare the differences between the different treatment groups (Figure 5A). In the process of observing the growth of the tumor, we noticed that the initial time of subcutaneous tumor formation in the interference group was significantly later than that in the other groups, and the tumor growth rate was also slower in the later stage (Figure 5C). Although the tumor volume of mice in the blank control group was slightly higher than that in the NC group, there was no significant difference between the NC group and the blank control group. In the picture, we can see that the volume of the blank control group seems to be much larger than that of the NC group, but the weight is only slightly larger than that of the NC group. This is because, although the blank control group looks larger, it is thinner. The reason why the weight of the overexpression group is much larger than that of the control group is that the tumor is thicker, which can be observed by the height of the protruding skin of the nude mice. The proliferation rate and the final tumor size of the overexpressed KIF2C group were undoubtedly larger than those of the other groups. After fixing each subcutaneous tumor with paraformaldehyde, the tumor was dried and weighed for statistical analysis (Figure 5B). When the mice were sacrificed, the average diameter of the control group was approximately 1.1 cm, while the overexpressed group reached 1.4 cm. However, in terms of tumor volume, the most obvious contrast was between the interference group and the control group. The tumor in the interference group was flatter, and the weight of the tumor was much smaller than that in the control group. This is consistent with the in vitro results. Meanwhile, we built a lung metastasis model by tail vein injection (Figure 5D). Eight weeks later, the samples of lung metastases were taken out, and it was observed with the naked eye that the lung surface of the sh-KIF2C group was smoother, and the number of tumor metastases was lower, while the lung surface of the KIF2C group was full of lesions (Figure 5E). We also observed the same results under the microscope after H&E (hematoxylin–eosin) staining of the lung-embedded sections (Figure 5F).

### 3.6. The Potential Mechanism of KIF2C in PDAC

**Figure 6:**
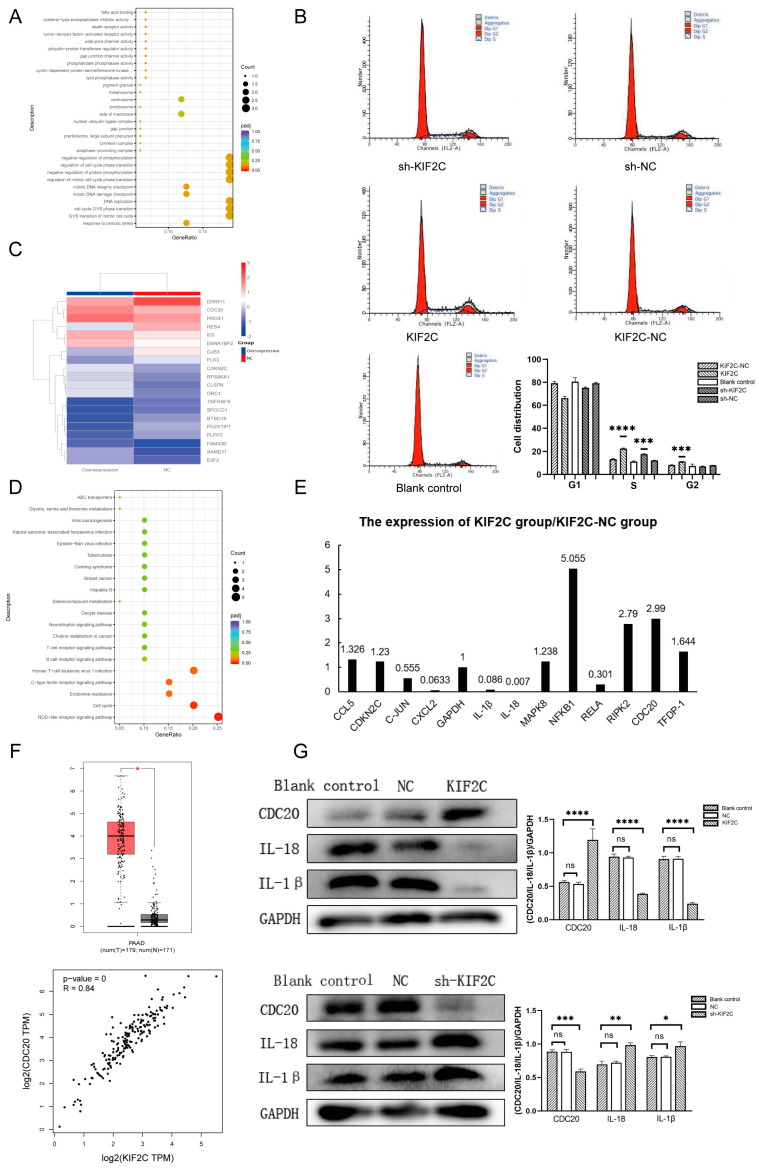
Analysis after sequencing and the results of cell cycle detection, real-time PCR, and Western blot validation. (**A**) The results of the GO analysis, in which the functions related to the cell cycle and mitosis are the most noteworthy. (**B**) Cell cycle detection of the transfected cell lines in each group, during which the S phase of the sh-KIF2C and KIF2C groups increased (*** *p* < 0.001, **** *p* < 0.0001). (**C**) Heat map of the differential genes in the overexpression and NC groups. (**D**) The results of the KEGG analysis, where the Nod-like receptor and cell cycle signaling pathways are perhaps the main pathways in which KIF2C plays a role. (**E**) The results of the real-time PCR detection of some differential genes, showing that changes in KIF2C do affect the expression of other genes. (**F**) Correlation analysis between KIF2C and CDC20 in the TCGA database (Red box and gray box represent cancer tissue and paracancerous tissue respectively). (**G**) Western blot analysis of the IL-1β, IL-18, and CDC20 levels (* *p* < 0.05, ** *p* < 0.01, *** *p* < 0.001, **** *p* < 0.0001).

After completing the experiments in vivo and in vitro, we have to think about how KIF2C has such an effect on pancreatic cancer cells. Therefore, we sent the transfected cells to be sequenced by Trizol cleavage in order to understand which genes changed and which signal pathways were affected when KIF2C changed. We performed the GO (Gene Ontology) analysis, differential gene screening, and KEGG analysis after obtaining the sequencing results. In the GO analysis, we learned that KIF2C is related to many functions, such as the mitotic DNA integrity checkpoint, response to osmotic stress, and DNA replication (Figure 6A). What interests us most out of these functions is the cell cycle, because KIF2C is a mitotic centromere-associated kinesin. Therefore, we detected the cell cycle of transfected pancreatic cancer by flow cytometry (Figure 6B). The results of the cell cycle detection showed that, compared to the control group, the S phase of the sh-KIF2C and KIF2C groups increased, while the S phase of the KIF2C group was longer. Combined with the results of the proliferation assays, we believe that the increase in the S phase in the KIF2C group is due to the abnormal activity of DNA synthesis, while, in the sh-KIF2C group, the S phase is prolonged due to the block of DNA synthesis in this phase. This is because the G2 phase increased after S phase prolongation in the KIF2C group, while the G2 phase did not increase in the sh-KIF2C group. In the differential gene screening, we found some genes that are statistically significant (Figure 6C). Similarly, we obtained several statistically significant signal pathways in the KEGG analysis (Figure 6D). Finally, we decided to select some differential genes in the pathway for verification. For these genes, we first carried out a preliminary detection by qPCR (Figure 6E). It is not difficult to see that the signal pathways with significant statistical differences are related to the cell cycle and immunity. After verifying the expression level of RNA, we selected three indicators from the two aspects of immunity and the cell cycle to verify the alteration of the protein level (Figure 6G). The results of the WB showed that the overexpression of KIF2C caused the downregulation of IL-1β and IL-18, and the expression of KIF2C was positively correlated with CDC20, which is consistent with the results of the correlation analysis in the TCGA database (Figure 6F).

## 4. Discussion

In this study, we first identified the high expression of KIF2C in pancreatic cancer by TCGA and subsequently verified this finding at the DNA and protein levels. After combining the expression level of KIF2C with the clinical information of patients, it was not difficult to find that the high expression level of KIF2C was positively correlated with the degree of malignancy of PDAC. Therefore, we speculate that KIF2C may play a crucial role in the occurrence and development of pancreatic cancer. To test this hypothesis, we designed a series of in vivo and in vitro assays to verify the effect of KIF2C on the degree of malignancy of PDAC. Compared to the control group, the tumor volume of KIF2C-overexpressing mice was larger, and the tumorigenesis time was shorter. In stark contrast, it significantly inhibited the growth of tumors in the shRNA group. Not only that, but we observed that KIF2C overexpression promoted tumor metastasis in a mouse lung tumor formation assay by tail vein injection, resulting in large areas of metastasis and the diffusion of cancer cells in the lungs of the mice. In recent years, the close relationship between KIF2C and different tumors has been gradually revealed, which coincides with our results [23,24,25]. Sacha et al. found that KIF2C is significantly overexpressed in colorectal and other epithelial cancers, and the proliferative activity of the tumor is correlated with KIF2C expression levels [25]. In fact, we are not surprised about these research findings due to the “background” of KIF2C.

**Figure 7:**
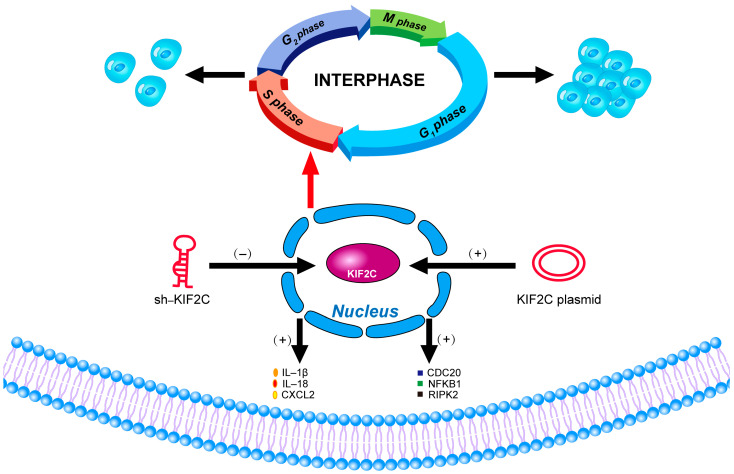
The impact of KIF2C alterations.

KIF2C is a member of the kinesin 13 family, which regulates the processes of spindle assembly, chromosome aggregation, and separation [12,13,26]. The main function of KIF2C is to regulate the dynamics of microtubules during mitosis. It is located in the centrosome, the centromere/centromere region, and the middle of the spindle [22,27,28]. As KIF2C recruits these regions, it participates in spindle assembly, correction of centromere–microtubule connection errors, and chromosome aggregation and separation [29,30,31]. Therefore, the inhibition or depletion of KIF2C disrupts normal spindle dynamics, resulting in abnormal chromosome aggregation and segregation and reducing the activity of KIF2C, especially in the centromeric region, causing damage to the movement of chromosomes [32,33,34,35]. Not only does KIF2C regulate the movement of chromosomes, but Zhu et al. also found that KIF2C is involved in DNA damage [36]. When we talk about DNA damage and the structural instability of chromosomes, we have to turn our attention to another focus—cancer [37,38,39]. Genomic instability is undoubtedly one of the main causes of tumorigenesis [40]. The evidence presented thus far supports the idea that KIF2C is closely related to the occurrence of cancer. Moreover, KIF2C may also play a crucial regulatory role in the immune microenvironment. Researchers in endometrial cancer have found that the knockout of KIF2C can inhibit the apoptosis of CD8+T cells and ki-67 expression [41]. In fact, KIF2C is involved in different signal pathways. For example, in cervical cancer, the downregulation of KIF2C can promote the activation of the p53 signal pathway [42]; in hepatocellular carcinoma, KIF2C promotes the development of hepatocellular carcinoma through the Ras/MAPK and PI3K/Akt signal pathways [43]. In this study, the results very clearly demonstrate that the higher the expression level of KIF2C, the higher the degree of malignancy of the tumor. Interference with KIF2C can effectively reduce the invasiveness of pancreatic cancer, which may become a promising target for the treatment of this cancer. In the last part of this paper, we verified the potential mechanism of KIF2C, showing that the interference and overexpression of KIF2C can affect the cell cycle of pancreatic cancer cells, which is also consistent with the bioinformatics enrichment analysis, while changes in KIF2C also cause some changes in the immune index and signal pathway (Figure 7). The results of the transcriptome suggest that KIF2C may be related to the phase transition of the cell cycle, which may be by way of a S phase change after KIF2C knockout or overexpression. In addition, we found that the expression of KIF2C was positively correlated with CDC20. As a key regulatory protein in the cell cycle, in recent years, CDC20 has been proven to promote the proliferation, migration, and invasion of pancreatic cancer [44,45,46]. CDC20 may also be involved in the role of KIF2C in pancreatic cancer. However, we did not conduct an in-depth exploration of the signal pathway of KIF2C’s function, revealing only the role of KIF2C in pancreatic cancer and the pathways and genes that may be related to it, which is also where this study needs to be improved.

## 5. Conclusions

In this study, we initially found a special expression of the KIF2C gene through bioinformatics and specimen detection. In the follow-up experiments, we clearly observed the effect of KIF2C on the proliferation and invasion of PDAC; the results of the sh-KIF2C group in the in vivo experiment were especially exciting. The downregulation of KIF2C can greatly inhibit the formation of subcutaneous tumors and lung metastasis. In the exploration of the potential mechanism, various results showed that KIF2C plays a regulatory role in the cell cycle, but this may not be the only way for KIF2C to promote the malignant proliferation of PDAC. The effect of KIF2C on pancreatic cancer may not only be limited to the development of PDAC but may also pull the “trigger” and cause tumorigenesis. At present, the therapeutic effect of pancreatic cancer is limited; we hope that the target of KIF2C can provide new ideas for its treatment, so that patients who cannot receive timely surgical treatment can obtain a better prognosis.

## Figures and Tables

**Table 1:** The relationship between KIF2C expression and the clinicopathological features of 70 patients with PDAC.

Clinicopathological Variables	*n*	KIF2C Expression		*p* Value
		Low/Moderate	High	
All cases	70	42	28	
Age (years)				
<60	32	18	14	
≥60	38	16	22	0.643
Gender				
Male	35	23	12	
Female	35	16	20	0.111
TNM stage				
IA	27	17	10	
IB	17	8	9	
IIA	0	0	0	
B	11	4	7	
III	0	0	0	
IV	14	4	10	0.028 (<0.05)
Differentiated degree				
Low/moderate	46	17	29	
High	24	17	7	0.022 (<0.05)
Drinking history				
Yes	21	12	9	
No	49	22	27	0.412
Obstructive jaundice				
Yes	39	17	22	
No	31	17	14	0.420

## Data Availability

Data are available upon request through the corresponding author.

## Supplementary Materials

The following supporting information can be downloaded at: https://www.mdpi.com/article/10.3390/cancers15051502/s1, File S1: Full Western blot images. File S2: The primer sequences.

## Abbreviations

PDAC: pancreatic ductal adenocarcinoma; IHC: immunohistochemistry; KIF2C: kinesin family member 2C; shRNA: small hairpin RNA; siRNA: small interfering RNA; TCGA: The Cancer Genome Atlas; IL-1β: interleukin-1β; IL-18: interleukin-18; CDC20: cell division cycle 20; GEPIA: Gene Expression Profiling Interactive Analysis; OS: overall survival; DFS: disease-free survival; GO: Gene Oncology; KEGG: Kyoto Encyclopedia of Genes and Genomes; NC: negative control; PAAD: pancreatic cancer.
