# Supplementary material for: KIF2C Facilitates Tumor Growth and Metastasis in Pancreatic Ductal Adenocarcinoma
Source: Cancers (Basel). 2023 Feb 27;15(5):1502. doi: 10.3390/cancers15051502 (PMC10000478; doi:10.3390/cancers15051502)

Supplementary File S1. Full Western blot images

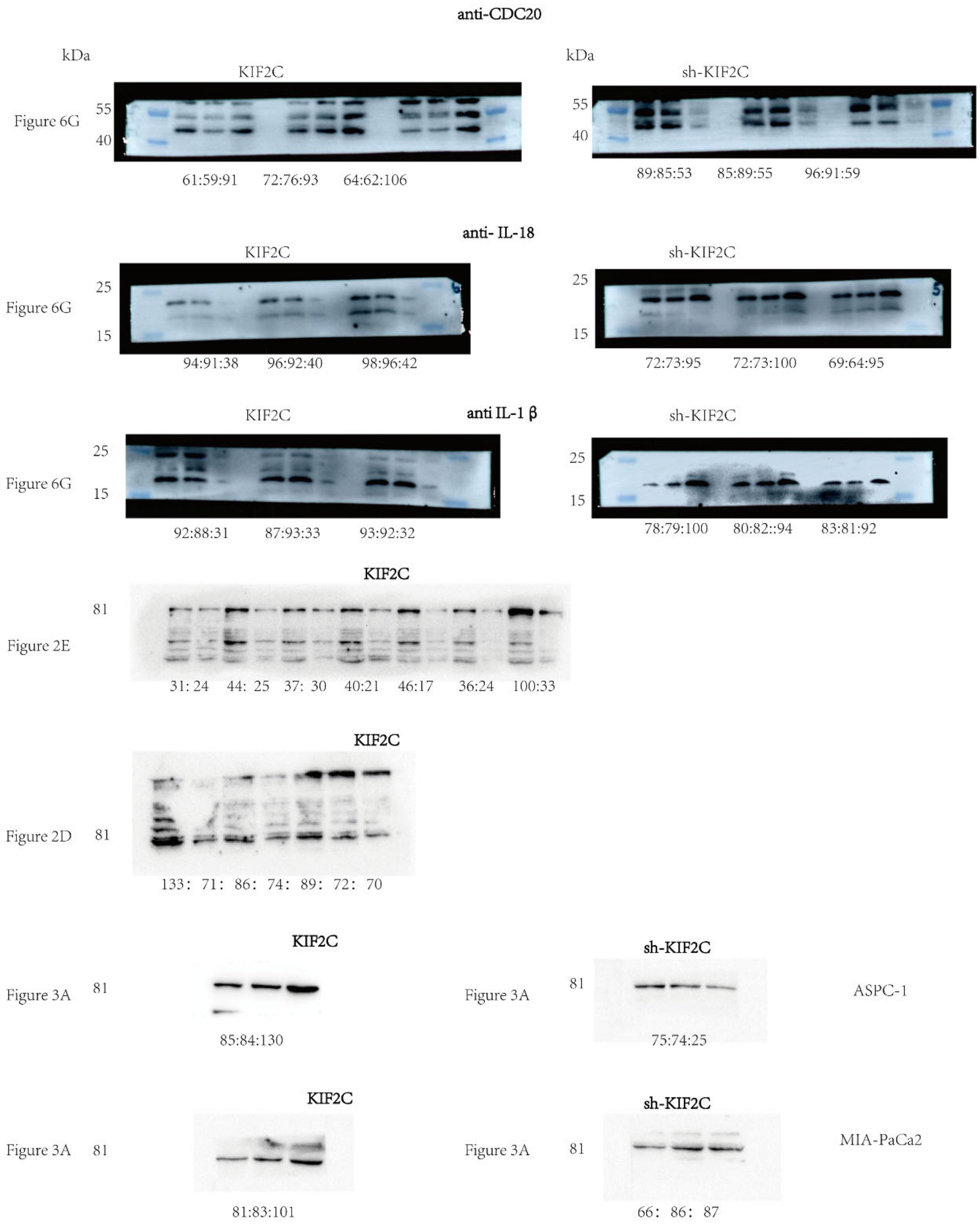

CDC20(55kDa) protein (sh-KIF2C). (This figure is the original image for Fig6G)

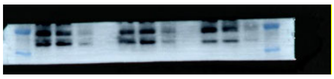

CDC20(55kDa) protein (KIF2C). (This figure is the original image for Fig6G)

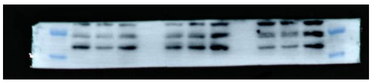

IL-18 (22kDa) protein (KIF2C). (This figure is the original image for Fig6G)

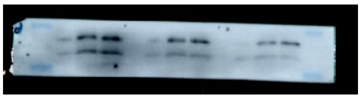

IL-18(22kDa) protein (sh-KIF2C). (This figure is the original image for Fig6G)

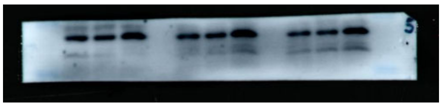

IL-1β(17kDa) protein （KIF2C）(This figure is the original image for Fig6G)

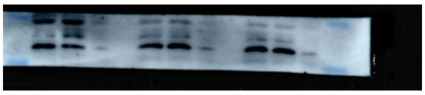

IL-1β(17kDa) protein（sh-KIF2C）(This figure is the original image for Fig6G)

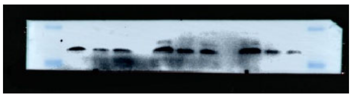

GAPDH (37kDa) (CDC20) (This figure is the original image for Fig6G)

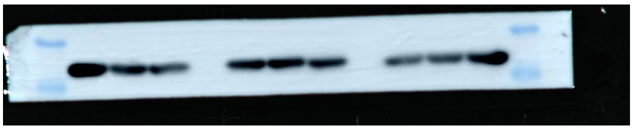

GAPDH(37 kDa) (IL-1β) (This figure is the original image for Fig6G)

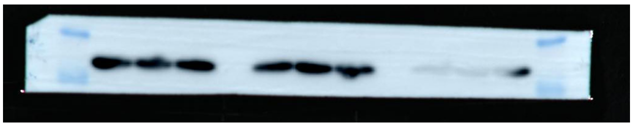

GAPDH (37kDa) (IL-18) (This figure is the original image for Fig6G)

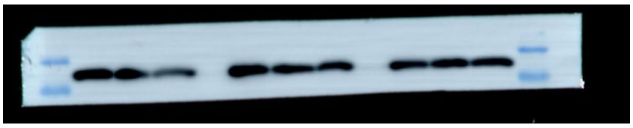

Transfection WB (KIF2C, 81 kDa)

ASPC-1 (KIF2C) ASPC-1 (sh-KIF2C) (This figure is the original image for Fig3A)

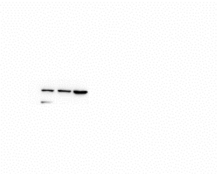
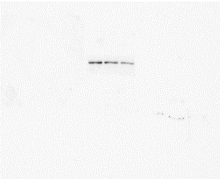

GAPDH

MIA-PaCa2 (KIF2C)   MIA-PaCa2 (sh-KIF2C) (This figure is the original image for Fig3A)

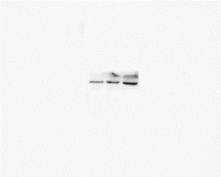

GAPDH

Seven Cell lines WB   GAPDH   (This figure is the original image for Fig2D)

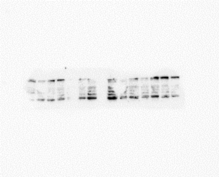

Tissue WB   GAPDH (This figure is the original image for Fig2E)

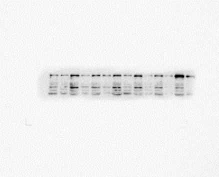
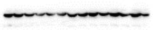

Supplement: Supplementary file 1 [file cancers-15-01502-s001.zip › cancers-2241046-Supplementary File S1.pdf]
